# Plasma Steroid Profiling Between Patients With and Without Diabetes Mellitus in Nonfunctioning Adrenal Incidentalomas

**DOI:** 10.1210/jendso/bvae140

**Published:** 2024-07-31

**Authors:** Yui Nakano, Maki Yokomoto-Umakoshi, Kohta Nakatani, Hironobu Umakoshi, Hiroshi Nakao, Masamichi Fujita, Hiroki Kaneko, Norifusa Iwahashi, Tatsuki Ogasawara, Tazuru Fukumoto, Yayoi Matsuda, Ryuichi Sakamoto, Yoshihiro Izumi, Takeshi Bamba, Yoshihiro Ogawa

**Affiliations:** Department of Medicine and Bioregulatory Science, Graduate School of Medical Sciences, Kyushu University, Fukuoka 812-8582, Japan; Department of Medicine and Bioregulatory Science, Graduate School of Medical Sciences, Kyushu University, Fukuoka 812-8582, Japan; Division of Metabolomics, Medical Research Center for High Depth Omics, Medical Institute of Bioregulation, Kyushu University, Fukuoka 812-8582, Japan; Department of Medicine and Bioregulatory Science, Graduate School of Medical Sciences, Kyushu University, Fukuoka 812-8582, Japan; Department of Medicine and Bioregulatory Science, Graduate School of Medical Sciences, Kyushu University, Fukuoka 812-8582, Japan; Department of Medicine and Bioregulatory Science, Graduate School of Medical Sciences, Kyushu University, Fukuoka 812-8582, Japan; Department of Medicine and Bioregulatory Science, Graduate School of Medical Sciences, Kyushu University, Fukuoka 812-8582, Japan; Department of Medicine and Bioregulatory Science, Graduate School of Medical Sciences, Kyushu University, Fukuoka 812-8582, Japan; Department of Medicine and Bioregulatory Science, Graduate School of Medical Sciences, Kyushu University, Fukuoka 812-8582, Japan; Department of Medicine and Bioregulatory Science, Graduate School of Medical Sciences, Kyushu University, Fukuoka 812-8582, Japan; Department of Medicine and Bioregulatory Science, Graduate School of Medical Sciences, Kyushu University, Fukuoka 812-8582, Japan; Department of Medicine and Bioregulatory Science, Graduate School of Medical Sciences, Kyushu University, Fukuoka 812-8582, Japan; Division of Metabolomics, Medical Research Center for High Depth Omics, Medical Institute of Bioregulation, Kyushu University, Fukuoka 812-8582, Japan; Division of Metabolomics, Medical Research Center for High Depth Omics, Medical Institute of Bioregulation, Kyushu University, Fukuoka 812-8582, Japan; Department of Medicine and Bioregulatory Science, Graduate School of Medical Sciences, Kyushu University, Fukuoka 812-8582, Japan

**Keywords:** steroid profiles, adrenal incidentalomas, diabetes mellitus, 11-oxygenated androgens, 11β-hydroxytestosterone

## Abstract

**Context:**

Adrenal incidentalomas, including nonfunctioning adrenal incidentalomas (NFAI), are associated with a high prevalence of diabetes mellitus (DM). While NFAI is diagnosed by exclusion when no hormone excess exists, subtle cortisol secretion may exist and contribute to DM development. However, it alone cannot explain the increased risk, and whether other steroid metabolites are involved remains unclear.

**Purpose:**

To investigate steroid metabolites associated with DM in patients with NFAI using plasma steroid profiles.

**Methods:**

Using liquid chromatography-tandem mass spectrometry, 22 plasma steroid metabolites were measured in 68 patients with NFAI (31 men and 37 women). Data were adjusted for age before normalization.

**Results:**

Discriminant analysis showed that plasma steroid profiles discriminated between patients with and without DM in men (n = 10 and = 21, respectively) but not women: 11β-hydroxytestosterone, an adrenal-derived 11-oxygenated androgen, contributed most to this discrimination and was higher in patients with DM than in those without DM (false discovery rate = .002). 11β-hydroxytestosterone was correlated positively with fasting plasma glucose (r = .507) and hemoglobin A1c (HbA1c) (r = .553) but negatively with homeostatic model assessment of β-cell function (HOMA2-B) (r = −.410). These correlations remained significant after adjusting for confounders, including serum cortisol after the 1-mg dexamethasone suppression test. Bayesian kernel machine regression analysis verified the association of 11β-hydroxytestosterone with HbA1c and HOMA2-B in men.

**Main Conclusion:**

Plasma steroid profiles differed between those with and without DM in men with NFAI. 11β-hydroxytestosterone was associated with hyperglycemia and indicators related to pancreatic β-cell dysfunction, independently of cortisol.

With the advancement of imaging diagnostic techniques, the opportunity to discover adrenal incidentalomas (AI) has increased; the prevalence of AI is approximately 3% in adults over 50 years old and up to 10% in patients over 80 years old [1]. The majority of AI are adrenocortical adenomas, and overt hormonal abnormalities are rare, but 30% to 50% exhibit mild autonomous cortisol secretion (MACS), and 40% to 70% are nonfunctioning adrenal incidentalomas (NFAI) [2]. While it is well-known that functioning adrenal tumors, including MACS, are frequently associated with cardiometabolic disorders, even patients with NFAI have been reported to have a higher prevalence of these disorders, such as hypertension and diabetes mellitus (DM), than those without adrenal tumors [3-6].

DM is a particularly important clinical issue among cardiometabolic disorders due to its severe complications, increased mortality risk, and economic burden from a healthcare perspective, with significant effects on patients' quality of life and prognosis [7, 8]. NFAI is associated with insulin resistance [5], and approximately 20% to 30% of the patients have concomitant DM [3, 4]. There are a few previous reports of low amounts of cortisol secretion in NFAI [9-11], and even subtle autonomous cortisol secretion in NFAI is associated with insulin resistance and the risk of DM [3, 5]. Indeed, no difference in the prevalence of DM between patients with NFAI and MACS has been reported [4, 12], suggesting that the risk of DM in NFAI cannot be explained by cortisol alone.

In recent years, steroid profiling, a comprehensive evaluation of steroid metabolites, including steroid intermediate metabolites, has been performed on patients with AI. This has provided insights into the diagnosis and pathophysiology of AI [9, 13-15]. We recently reported that multiple adrenal-derived steroid metabolites are associated with bone strength in patients with AI and hypercortisolemia, and their pathophysiological significance in metabolic disorders has been elucidated [16]. We hypothesized that steroid metabolites other than cortisol are involved in the development of DM in patients with NFAI. However, the steroid profiles of patients with NFAI and DM have not been well characterized. In this study, we aimed to elucidate steroid metabolites associated with DM in patients with NFAI using plasma steroid profiles.

## Materials and Methods

### Study Design

This retrospective cross-sectional study was conducted at Kyushu University Hospital, a referral center in Japan. The study protocol was approved by the Institutional Ethics Committee (No. 21025-03). The study was performed in accordance with the guidelines for clinical studies published by the Ministry of Health, Labour and Welfare, Japan. Informed consent was obtained from all the patients.

### Inclusion and Exclusion Criteria

**Figure 1.**
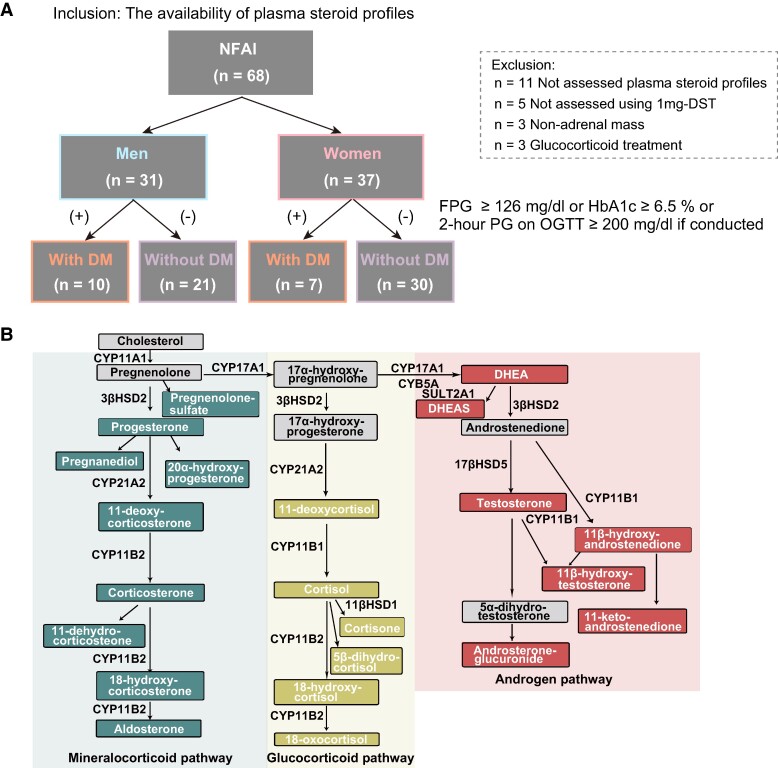
Study design and steroid pathways. (A) The flowchart depicts the process of patient selection for the study. (B) Steroid pathways of mineralocorticoids, glucocorticoids, and androgens are shown, and the steroid metabolites used in this study are highlighted.

Abbreviations: DHEA, dehydroepiandrosterone DHEA-S, dehydroepiandrosterone-sulfate; DM, diabetes mellitus; DST, dexamethasone suppression test; FPG, fasting plasma glucose; HbA1c hemoglobin A1c; NFAI, nonfunctioning adrenal incidentaloma.

This study included consecutive patients with NFAI who were admitted to our endocrine unit between January 2013 and September 2021. The inclusion criterion was the availability of plasma steroid profiles. The exclusion criteria were as follows: patients without a plasma steroid profile assessment (n = 11), patients who did not undergo the 1-mg dexamethasone suppression test (DST; n = 5), patients without an adrenal mass on computed tomography (n = 3), and patients receiving glucocorticoid treatment (n = 3). Consequently, 68 patients with NFAI were included in this study (Fig. 1A).

### Diagnosis and Data Collection

AI was defined as adrenal tumors incidentally discovered for reasons other than suspected adrenal disease. NFAI was defined as those with serum cortisol after 1-mg DST (hereafter referred to as DST cortisol) below 1.8 μg/dL, according to the guidelines of the European Society of Endocrinology [1], and not diagnosed as primary aldosteronism, pheochromocytomas, or adrenocortical carcinomas based on clinical data, imaging findings, or postoperative pathological findings. DM was diagnosed when the patient had hemoglobin A1c (HbA1c; National Glycohemoglobin Standardization Program) ≥ 6.5%, a fasting plasma glucose level (FPG) ≥ 126 mg/dL, or 2-hour PG on oral glucose tolerance test (OGTT) ≥ 200 mg/dL if conducted, according to the guidelines of the American Diabetes Association [17]. Alcohol intake was defined as >3 units/day and smoking status was defined as past or present. Menopause was defined as the absence of menstruation for at least one year at baseline, and the data were obtained via an interviewer-assisted questionnaire.

### Biochemical Measurements and Assay Methods

We collected basal serum cortisol at 0800 hours (reference range, 6.2–18.0 μg/dL), midnight serum cortisol at 2300 hours, morning plasma adrenocorticotropic hormone (ACTH), and DST cortisol. For DST cortisol, 1 mg of dexamethasone was administered at 2300 hours the day before sample collection. Reference ranges and assay methods have been described previously [18]. HbA1c and estimated glomerular filtration rate (eGFR) were measured, as previously described [18]. Homeostatic model assessment of β-cell function (HOMA2-B) and insulin resistance (HOMA2-IR) was calculated from FPG and serum C-peptide data with the HOMA2 Calculator, version 2.2.4 (Diabetes Trials Unit, University of Oxford, Oxford, UK) [19, 20].

### Plasma Steroid Profiling

Morning plasma samples were collected and centrifuged after overnight fasting. The supernatant was stored in a deep freezer (−80 °C) until analysis. We quantified 22 plasma steroid metabolites using liquid chromatography-tandem mass spectrometry (Fig. 1B), as previously described [16, 21]. Plasma steroid profiles are also influenced by age and sex [22]. Therefore, we analyzed the data separately for men and women. Additionally, we adjusted for the effect of age by incorporating it into the linear regression model and analyzing the resulting residuals [16, 23]. The missing values were imputed as one-fifth of the minimum positive value. Data were then normalized to the median and log-transformed using MetaboAnalyst 6.0 [24]. All subsequent downstream analyses were performed using the normalized datasets.

Supervised partial least squares-discriminant analysis (PLS-DA) was performed to determine whether plasma steroid profiles could discriminate between patients with and without DM. PLS-DA is a statistical method used to identify differences between groups (in this case, patients with and without DM) based on multiple variables (steroid metabolites) [25]. Variable importance in the projection (VIP) score analysis was performed, which ranked the overall contribution of each variable to the PLS-DA model. VIP scores > 1 were considered statistically significant. Cliff's delta value is a measure of effect size that quantifies the degree of overlap between 2 groups of observations. It ranges from −1 to 1, where 0 indicates complete overlap and −1 or 1 indicates no overlap between the groups [26]. In our study, it helps to assess the magnitude of differences in steroid metabolite levels between patients with and without DM. Their statistical significance was tested using the Brunner–Munzel test, indicating suggestive significance at *P* < .05 and statistical significance at a false discovery rate (FDR) < .1. For steroid metabolites exhibiting suggestively or statistically significant differences in Cliff's delta values, we conducted an analysis of covariance after adjusting for possible confounders, including body mass index (BMI), eGFR, alcohol intake, smoking status, and DST cortisol.

To investigate the relationship between steroid metabolites and glycemic control measures, we evaluated the correlation of each steroid metabolite adjusted for age with FPG and HbA1c levels using Spearman's rank correlation coefficient. For steroid metabolites that showed suggestive (*P* < .05) or statistically significant (FDR < .1) correlations with either FPG or HbA1c, we further assessed their correlations with HOMA2-B and HOMA2-IR to explore potential associations between steroid metabolites and insulin secretion or insulin resistance. Spearman's partial correlation coefficient between the relevant steroid metabolites and glycemic control measures was then assessed, controlling for possible confounders such as BMI, eGFR, alcohol intake, smoking status, and DST cortisol.

Bayesian kernel machine regression (BKMR) analysis was employed to assess the relative contributions of steroid metabolites of interest to glycemic control measures while accounting for potential nonlinear relationships and interactions between the metabolites [27]. BKMR allows for the estimation of posterior inclusion probabilities (PIPs), which quantify the importance of each steroid metabolite by measuring the probability that a variable is included in the model-given data. This approach enabled the identification of the most relevant steroid metabolites associated with glycemic control measures while adjusting for relevant covariates, including BMI, eGFR, alcohol intake, smoking status, and DST cortisol. Hierarchical variable selection was performed with 10 000 iterations via the Markov chain Monte Carlo algorithm using the R package “bkmr” [28].

### Statistical Power Analysis

We conducted a priori statistical power analyses for our 2 main analyses using the R package “pwr” (version 1.3.0): (1) the comparison of each steroid metabolite between patients with and without DM and (2) the correlation of steroid metabolites with glycemic control measures. For analysis (1), we assumed an effect size of .8 (Cohen's d) and set the significance level at .05. The resulting power was .521 for men and .457 for women. For analysis (2), we assumed an effect size with a correlation coefficient of .5 and set the significance level at .05, which yielded a power of .838 for men and .899 for women.

### Statistics

Statistical analyses were performed using the R software (version 4.2.2). The Mann–Whitney U-test and Fischer's exact test were used to examine the baseline characteristics of patients with and without DM, as appropriate. Statistical tests were 2-tailed, and statistical significance was set at *P* < .05 unless otherwise stated.

## Results

### Baseline Characteristics of Patients With and Without DM in NFAI

Baseline characteristics were evaluated in 68 patients with NFAI (Table 1). Among the men (n = 31), 10 (32.3%) had DM and 21 (67.7%) did not. Among the women (n = 37), 7 (18.9%) had DM and 30 (81.1%) did not; the prevalence of DM in patients with NFAI observed in this study is roughly comparable to that in previous reports [3-5]. In NFAI, patients with DM had higher FPG and HbA1c levels than those without DM, regardless of sex. Men with DM had higher levels of serum C-peptide and HOMA2-IR than those without DM, whereas women with DM had lower levels of HOMA2-B than those without DM. In addition, in men with NFAI, patients with DM had higher levels of DST cortisol (median [interquartile ranges]; 1.3 [1.2, 1.4] μg/dL and .8 [.7, 1.1] μg/dL; *P* = .006) and midnight cortisol (4.5 [3.4, 5.7] μg/dL and 2.6 [1.9, 3.0] μg/dL; *P* = .006) than those without DM, which is consistent with previous reports [3-5].

**Table 1.** Baseline characteristics of study patients

	Men		*P*-value	Women		*P*-value
	Without DM	With DM		Without DM	With DM	
No. of patients	21	10	—	30	7	
Age, y	56.0 [50.0, 67.0]	61.0 [54.2, 68.8]	.434	57.5 [52.5, 69.0]	67.0 [60.5, 71.0]	.145
Menopausal status, postmenopausal women %	—	—	—	83.3 (25/30)	100 (7/7)	.584
BMI, kg/m^2^	26.5 [22.7, 27.6]	26.1 [24.8, 27.3]	.642	23.2 [21.4, 27.0]	27.0 [23.8, 30.2]	.068
Alcohol intake, %	42.9 (9/21)	60% (6/10)	.611	43.3 (13/30)	14.3 (1/7)	.320
Smoking status, %	38.1 (8/21)	70% (7/10)	.202	43.3 (13/30)	28.6 (2/7)	.773
FPG mg/dL	99.0 [92.0, 103.0]	126.0 [106.2, 140.5]	<.001*^a^*	91.5 [85.2, 100.0]	120.0 [112.0, 127.5]	.001*^a^*
HbA1c, %	5.7 [5.4, 6.0]	6.6 [6.3, 7.2]	.001*^a^*	5.8 [5.5, 6.0]	6.6 [6.4, 7.2]	<.001*^a^*
CPR, ng/mL	2.0 [1.6, 2.5]*^b^*	2.6 [2.4, 2.8]*^b^*	.048*^a^*	2.0 [1.2, 2.2]*^c^*	2.1 [1.8, 2.5]	.311
HOMA2-B	104.8 [94.2, 128.1]*^b^*	87.5 [73.2, 108.5]*^b^*	.114	106.2 [87.2, 122.0]*^c^*	82.4 [65.7, 85.8]	.021*^a^*
HOMA2-IR	1.6 [1.2, 1.9]*^b^*	2.0 [1.8, 2.3]*^b^*	.048*^a^*	1.5 [.9, 1.7]*^c^*	1.7 [1.4, 1.9]	.149
SBP, mmHg	137.0 [123.0, 144.0]	142.0 [137.2, 147.5]	.262	130.0 [119.0, 136.5]	129.0 [125.5, 139.5]	.684
DBP, mmHg	81.0 [74.0, 92.0]	87.0 [82.5, 93.5]	.228	84.5 [75.2, 95.0]	74.0 [70.0, 79.5]	.046*^a^*
TG, mg/dL	149.0 [122.0, 242.0]	175.0 [164.0, 195.0]	.735	120.0 [87.8, 151.8]	128.0 [121.0, 211.0]	.092
HDL-C, mg/dL	43.0 [36.0, 50.0]	51.5 [40.8, 57.2]	.320	54.5 [44.2, 67.8]	41.0 [37.5, 48.0]	.033*^a^*
LDL-C, mg/dL	111.0 [91.0, 135.0]	104.0 [86.5, 118.5]	.526	106.5 [99.0, 123.5]	108.0 [93.0, 111.5]	.548
eGFR, mL/min/1.73m^2^	86.0 [74.0, 97.0]	82.7 [65.8, 93.8]	.735	76.0 [70.3, 82.3]	82.0 [59.7, 89.0]	.892
Maximum tumor size on CT, mm	18.0 [10.0, 25.0]	24.0 [18.2, 39.5]	.176	16.0 [13.0, 22.8]	15.0 [12.5, 20.5]	.907
ACTH, pg/mL	32.8 [24.8, 50.9]	21.9 [18.3, 31.8]	.099	20.9 [13.6, 26.5]	17.4 [7.8, 24.1]	.194
Serum cortisol, μg/dL	12.4 [9.5, 15.3]	12.8 [9.6, 16.8]	.486	10.6 [8.2, 16.8]	7.5 [6.4, 11.6]	.107
DST cortisol, μg/dL	.8 [.7, 1.1]	1.3 [1.2, 1.4]	.006*^a^*	1.1 [.8, 1.4]	1.4 [.9, 1.6]	.234
Serum midnight cortisol, μg/dL	2.6 [1.9, 3.0]	4.5 [3.4, 5.7]	.006*^a^*	3.2 [2.0, 4.8]	2.8 [2.5, 3.5]	.548

Data are expressed as median [interquartile range] or percentage (number of patients).

^*a*^ *P* < .05 was considered significant.

^*b*^Missing 1 value.

^*c*^Missing 2 values.

Abbreviations: BMI, body mass index; CPR, C-peptide; CT, computed tomography; DBP, diastolic blood pressure; DM, diabetes mellitus; DST, dexamethasone suppression test; eGFR, estimated glomerular filtration rate; FPG, fasting plasma glucose; HbA1c, hemoglobin A1c; HDL-C, high-density lipoprotein cholesterol; HOMA2-B, homeostatic model assessment 2-B; HOMA2-IR, homeostatic model assessment 2-insulin resistance; LDL-C, low-density lipoprotein cholesterol; SBP, systolic blood pressure; TG, triglyceride.

### Plasma Steroid Profiles of Patients With and Without DM in NFAI

**Figure 2.**
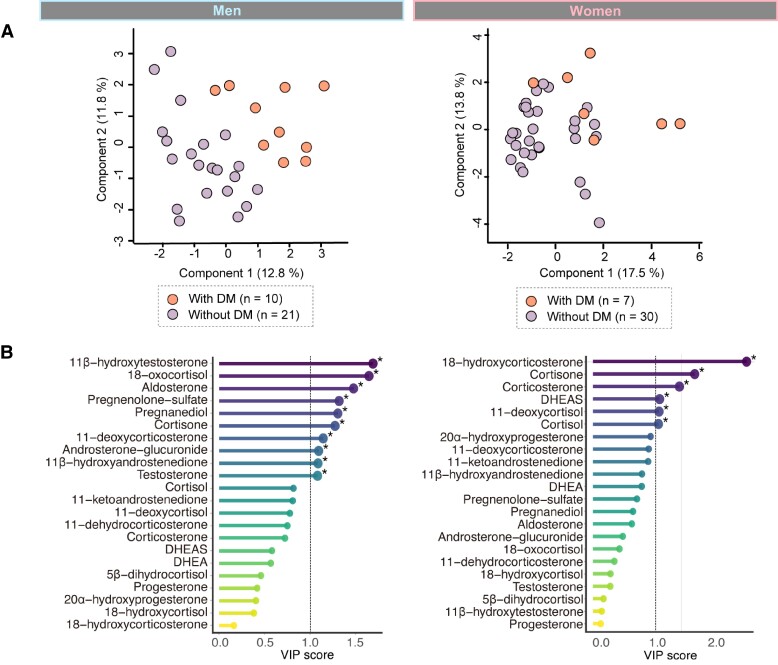
Plasma steroid profiles of patients with and without DM in NFAI. (A) PLS-DA adjusted for age was performed to determine whether plasma steroid profiles discriminate between patients with DM and those without DM in NFAI. Dots represent individual samples. (B) A VIP score analysis was performed to rank the overall contribution of each variable to the PLS-DA model. *VIP score > 1 was considered significant. The dot plots show the VIP score of component 1 of the PLS-DA model. The analysis was categorized into men (with DM, n = 10; without DM, n = 21) and women (with DM, n = 7; without DM, n = 30).

Abbreviations: DM, diabetes mellitus; NFAI, nonfunctioning adrenal incidentaloma; PLS-DA, partial least squares-discriminant analysis; VIP, variable importance in projection.

To determine whether plasma steroid profiles could discriminate between patients with and without DM in NFAI, PLS-DA adjusted for age was performed (Fig. 2A). Plasma steroid profiles discriminated between patients with and without DM in men with NFAI (n = 10 and 21, respectively; R2 = .54, Q2 = .15, and *P* for 1000 permutations = .04). In the VIP score analysis, 10 steroid metabolites contributed to the discrimination between the 2 groups of men with NFAI, with 11β-hydroxytestosterone ranking the highest (VIP score = 1.688) (Fig. 2B and Supplementary Table S1) [29]. In contrast, in women with NFAI, plasma steroid profiles did not clearly discriminate between patients with and without DM (n = 7 and n= 30, respectively).

### Comparison of Each Steroid Metabolite Between Patients With and Without DM in NFAI, Stratified by Sex

**Figure 3.**
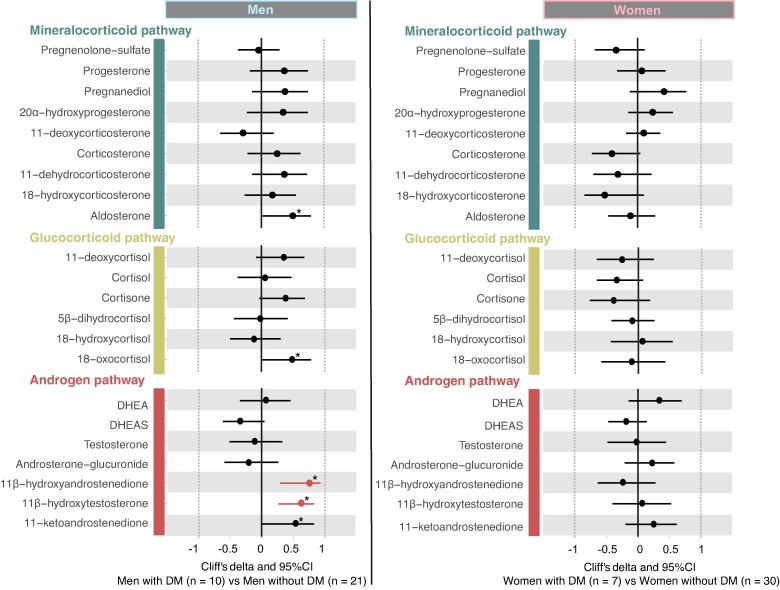
Comparison of each steroid metabolite between patients with and without DM in NFAI, stratified by sex. Cliff's delta values adjusted for age were evaluated to compare each steroid metabolite between patients with DM and without DM in NFAI. The figure is divided into 2 main panels: the left panel for men and the right panel for women. In each panel, we compare steroid metabolite levels between patients with DM and without DM within the same sex group. The forest plots of Cliff's delta and 95% CI; asterisks denote *P* < .05, and color highlights indicate FDR < .1, with red showing higher levels and blue showing lower levels in patients with DM. Statistical significance was tested using the Brunner–Munzel test, indicating suggestive significance at *P* < .05 and statistical significance at FDR < .1. For men, the comparison is between 10 patients with NFAI and DM vs 21 patients with NFAI without DM. For women, the comparison is between 7 patients with NFAI and DM vs 30 patients with NFAI without DM.

Abbreviations: CI, confidence interval; DM, diabetes mellitus; FDR, false discovery rate; NFAI, nonfunctioning adrenal incidentaloma.

To compare each steroid metabolite between patients with and without DM in the NFAI, we evaluated Cliff's delta values adjusted for age (Fig. 3 and Supplementary Table S2) [29]. In men with NFAI, patients with DM (n = 10) showed significantly higher levels of 2 steroid metabolites, 11β-hydroxyandrostenedione (Cliff's delta = .752; 95% confidence interval = .283, .931, *P* = .0004, FDR = .002) and 11β-hydroxytestosterone (Cliff's delta = .629; 95% confidence interval = .259, .838, *P* = .0003, FDR = .002) than those without DM (n = 21). Three levels of other steroid metabolites (11-ketoandrostenedione, aldosterone, and 18-oxocortisol) were also suggestively higher in patients with DM than in those without DM (*P* < .05). For steroid metabolites exhibiting suggestively or statistically significant differences in Cliff's delta values, we conducted an analysis of covariance after adjusting for possible confounders (Supplementary Table S3) [29]. After adjusting for BMI, eGFR, alcohol intake, smoking status, and DST cortisol, 11β-hydroxytestosterone remained significantly different between patients with and without DM (*P* = .037). In contrast, the associations between the other 4 steroid metabolites and DM were attenuated after adjusting for confounders (*P* > .05). In women with NFAI, no significant differences in steroid metabolite levels were observed between the 2 groups (n = 7 and n = 30, respectively).

### Correlation of Steroid Metabolites With Glycemic Control Measures in Patients With NFAI

**Figure 4.**
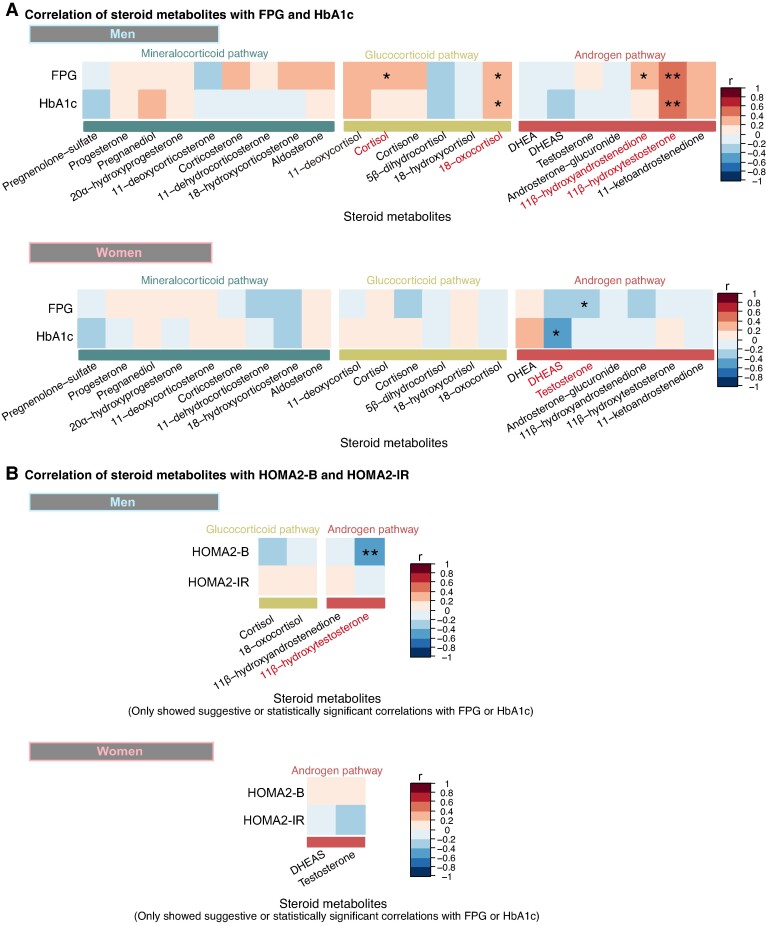
Correlation of steroid metabolites with glycemic control measures in patients with NFAI. (A) The correlations of each steroid metabolite adjusted for age with FPG and HbA1c were evaluated in men (n = 31) and women (n = 37). (B) For steroid metabolites that showed suggestive or statistically significant correlations with either FPG or HbA1c, we further assessed their correlations with HOMA2-B and HOMA2-IR in men (n = 29) and women (n = 35). The heatmaps display the Spearman's rank correlation coefficient. The row labels explicitly indicate steroid metabolites, which are grouped and color-coded by metabolic pathways. Metabolites showing suggestive or statistically significant correlations are highlighted in red text. The column labels represent glycemic control measures. Individual legends are provided for each correlation matrix: the color intensity indicates the strength of the correlation (r), with darker red indicating stronger positive correlations and darker blue indicating stronger negative correlations. *P < .05 was considered suggestively significant; **FDR < .1 was considered statistically significant.

Abbreviations: FDR, false discovery rate; FPG, fasting plasma glucose; HbA1c, hemoglobin A1c; HOMA2-B, homeostatic model assessment 2-B; HOMA2-IR homeostatic model assessment 2-insulin resistance; NFAI, nonfunctioning adrenal incidentaloma;

To investigate the relationship between steroid metabolites and glycemic control measures, we evaluated the correlation of each steroid metabolite adjusted for age with FPG and HbA1c levels using Spearman's rank correlation coefficients (Fig. 4A and Supplementary Table S4) [29]. In men with NFAI (n = 31), 11β-hydroxytestosterone exhibited significant positive correlations with both FPG (r = .507, *P* = .003, FDR = .025) and HbA1c (r = .553, *P* = .001, FDR = .008). In addition, 18-oxocortisol showed suggestive positive correlations with both FPG and HbA1c. In contrast, cortisol and 11β-hydroxyandrostenedione showed suggestive positive correlations with FPG only, although these associations did not reach statistical significance after FDR correction. In women with NFAI (n = 37), dehydroepiandrosterone sulfate (DHEA-S) exhibited a suggestive negative correlation with HbA1c, and testosterone showed a suggestive negative correlation with FPG, but these associations were not statistically significant. For steroid metabolites that showed suggestive or statistically significant correlations with either FPG or HbA1c, we further assessed their correlations with HOMA2-B and HOMA2-IR (Fig. 4B and Supplementary Table S4) [29]. In men with NFAI (n = 29), 11β-hydroxytestosterone had a significant negative correlation with HOMA2-B (r = −.410, *P* = .027, FDR = .054). Given that 11β-hydroxytestosterone was the only steroid metabolite showing statistically significant correlations with multiple glycemic control measures, we focused on this steroid metabolite in further analyses. In men with NFAI, 11β-hydroxytestosterone showed significant correlations, including a positive correlation with FPG (n = 31, estimate = .529, *P* = .006) and HbA1c (n = 31, estimate = .619, *P* = .001) and a negative correlation with HOMA2-B (n = 29, estimate = −0.475, *P* = .021) even after adjusting for BMI, eGFR, alcohol intake, smoking status, and DST cortisol in the Spearman's partial correlation coefficient (Supplementary Table S5) [29].

### Association Between Steroid Metabolites and Glycemic Control Measures in Men With NFAI

**Figure 5.**
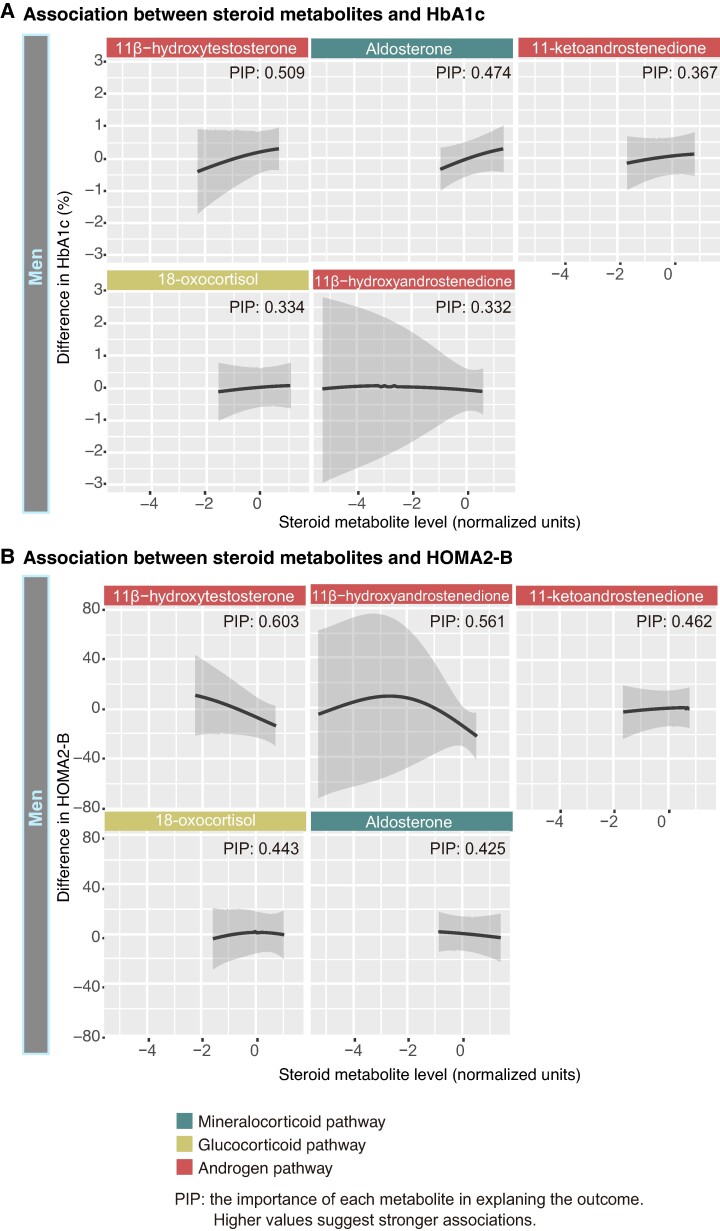
Association between steroid metabolites and glycemic control measures in men with NFAI. (A, B) BKMR analysis was performed to assess the relative contributions of 11β-hydroxytestosterone to glycemic control measures in men with NFAI. The association of single steroid metabolites with HbA1c (n = 31) or HOMA2-B (n = 29) were evaluated, holding all other steroid metabolites at the median. In this analysis, we focused on the 5 steroid metabolites (11β-hydroxytestosterone, 11β-hydroxyandrostenedione, 11-ketoandrostenedione, aldosterone, and 18-oxocortisol) that showed suggestive or statistically significant differences between patients with and without DM and covariates were defined as BMI, eGFR, alcohol intake, smoking status, and DST cortisol. The univariate dose-response function (solid line) and 95% CI (shaded areas) are shown for each metabolite. PIP values, indicating the importance of each metabolite in explaining the outcome, are displayed within each graph. Steroid pathways are color-coded as shown in the legend.

Abbreviations: BKMR, Bayesian kernel machine regression; BMI, body mass index; CI, confidence interval; DST, dexamethasone suppression test; eGFR, estimated glomerular filtration rate; HbA1c, hemoglobin A1c; HOMA2-B, homeostatic model assessment 2-B; NFAI, nonfunctioning adrenal incidentaloma; PIPs, posterior inclusion probabilities.

Furthermore, in men with NFAI, BKMR analysis was performed to assess the relative contributions of 11β-hydroxytestosterone to glycemic control measures (Fig. 5). In this analysis, we focused on the 5 steroid metabolites (11β-hydroxytestosterone, 11β-hydroxyandrostenedione, 11-ketoandrostenedione, aldosterone, and 18-oxocortisol) that showed suggestive or statistically significant differences between patients with and without DM and evaluated the association of them with HbA1c and HOMA2-B, with covariates including BMI, eGFR, alcohol intake, smoking status, and DST cortisol. In the BKMR analysis, 11β-hydroxytestosterone had the highest relative contribution to HbA1c (PIP = .509) and HOMA2-B (PIP = .603) among the 5 steroid metabolites, with linear positive and negative associations, respectively.

## Discussion

In this study, we aimed to identify the steroid metabolites associated with DM in patients with NFAI by comprehensively analyzing their plasma steroid profiles. We demonstrated that the plasma steroid profiles were clearly differentiated between patients with and without DM, particularly men. Notably, 11β-hydroxytestosterone, 1 of the adrenal-derived 11-oxygenated androgens, contributed the most to this discrimination and was higher in the DM group, independently of autonomous cortisol secretion. NFAI is currently defined as an AI that does not exhibit excessive secretion of measurable steroid hormones such as cortisol and aldosterone and is often diagnosed by exclusion when there is no evidence of hormone excess [1, 2]. On the other hand, our results suggest that, among AI currently defined as “nonfunctioning,” an imbalance of adrenal-derived steroid metabolites, not limited to cortisol, may be involved in the pathophysiology of metabolic disorders such as DM. These findings highlight the necessity of redefining “nonfunctioning,” considering the potential role of steroid intermediate metabolites in the development of metabolic disorders.

The mechanism by which 11β-hydroxytestosterone contributes to the development of DM in patients with NFAI remains to be elucidated. In the adrenal cortex, 11β-hydroxyandrostenedione and 11β-hydroxytestosterone, which are 11-oxygenated metabolites of androstenedione and testosterone, respectively, are under the control of cytochrome P450 11B1. These metabolites are subsequently converted to 11-ketoandrostenedione and 11-ketotestosterone in the peripheral tissues [30, 31]. ACTH regulates the production of these 11-oxygenated androgens [32]. Aging and chronic stress can activate the hypothalamic-pituitary-adrenal axis and stimulate ACTH secretion [33]. Furthermore, the boundary between the zona fasciculata and zona reticularis of the adrenal cortex becomes obscure (the 2-layered structure of zonae fasciculata and reticularis of the adrenal cortex becomes disrupted), thereby increasing the interface between cytochrome b5- and 3β-hydroxysteroid dehydrogenase type 2-expressing areas, which promotes the conversion of dehydroepiandrosterone to androstenedione [34]. These factors may contribute to increased 11-oxygenated androgen production. This hypothesis is supported by the previous reports that 11-oxygenated androgen levels show little change with age [34, 35], suggesting that in chronic stress conditions such as DM, 11-oxygenated androgens may increase in an ACTH-mediated manner. However, in the present study, we found no difference in ACTH levels between patients with and without DM. One possible explanation is the autonomous production of 11-oxygenated androgens by the adrenal tumors. Indeed, recent studies suggested ACTH-independent production of 11-oxygenated androgens in adrenal tumors [36, 37]. Further studies of intratumor steroid production are required to elucidate this mechanism.

We showed positive correlations of 11β-hydroxytestosterone with FPG and HbA1c in men with NFAI. These correlations remained significant even after adjusting for confounders, including DST cortisol, and consistent results were observed in the BKMR analysis. This finding suggests that, in addition to cortisol, 11β-hydroxytestosterone is involved in the progression of DM in NFAI. Although 11β-hydroxytestosterone has a weak binding affinity to the androgen receptor and is not thought to exert significant androgen effects on its own, it can indirectly exert androgen effects by being metabolized into 11-ketotestosterone, which has potent androgen activity in peripheral tissues [30, 31]. Indeed, 11-oxygenated androgens are the predominant androgens in polycystic ovary syndrome and are associated with a high rate of metabolic disorders due to hyperandrogenemia. These 11-oxygenated androgens have been shown to affect obesity and insulin sensitivity [38, 39]. Moreover, a recent study reported that hyperglycemia in polycystic ovary syndrome is not only associated with insulin resistance but also with dysfunction of pancreatic β-cells [40]. This finding is consistent with previous animal studies showing that androgen excess causes pancreatic β-cell dysfunction by inducing systemic oxidative stress [41, 42]. In this study, 11β-hydroxytestosterone was found to be negatively correlated with HOMA2-B, an index of pancreatic β-cell dysfunction. Taken together, these observations suggest that 11β-hydroxytestosterone contributes to hyperglycemia through dysfunction of pancreatic β-cells.

In this study, we observed sex-specific associations between plasma steroid profiles and DM in patients with NFAI. However, it is important to note that the small sample size, particularly the limited number of women with DM, may have contributed to the observed sex differences. This sample size constraint should be considered when interpreting our results. With this caveat in mind, our findings suggest the involvement of sex-specific adrenal-derived androgens. Specifically, an association between 11β-hydroxytestosterone and hyperglycemia was observed in men but not in women. Instead, our findings suggest that a reduction in DHEA-S, a significant adrenal androgen, may be associated with hyperglycemia in women. Circulating levels of 11β-hydroxytestosterone have been reported to be similar in men and women throughout adult life stages [34]. In contrast, DHEA-S is an essential source of androgens, particularly in women [43], and its reduction has been reported to be associated with reduced insulin secretion and sensitivity [44-46]. One possible explanation for the sex differences observed in this study could be that, in women with NFAI, the effect of reduced DHEA-S on glucose metabolism was more pronounced, relatively diminishing the influence of 11β-hydroxytestosterone.

This study has several limitations. First, it was conducted at a single center and was retrospective in nature. We acknowledge that our cross-sectional study design makes it challenging to establish causal relationships. While we found a significant correlation between 11β-hydroxytestosterone and glycemic control measures in men with NFAI and validated this association using BKMR analysis, it is important to interpret these findings cautiously. Correlation does not imply causation. The observed relationships may be due to underlying physiological mechanisms affecting both steroid metabolism and glucose homeostasis simultaneously or unmeasured confounding factors [47]. The directionality of these associations also cannot be definitively established from our data. Future longitudinal and experimental studies are needed to elucidate potential causal relationships. Second, the relatively small sample size is a limitation. Our study was adequately powered for detecting correlations of steroid metabolites with glycemic control measures. However, for both men and women with NFAI, it was underpowered for detecting differences in steroid metabolites between patients with and without DM, with power below the conventionally desired level of .80. This indicates a possibility of type II error. Therefore, our negative findings should be interpreted with caution. Future studies with larger sample sizes are needed to detect potentially smaller effect sizes with greater reliability and to confirm our findings. Furthermore, in this study, pancreatic β-cell dysfunction was assessed by HOMA2-B alone. This study may not have detected functional impairments in the early stages of glucose intolerance, which needs to be evaluated and validated using oral glucose and glucagon tolerance tests.

This study showed that the plasma steroid profiles clearly discriminate between patients with and without DM in men with NFAI; 11β-hydroxytestosterone was associated with hyperglycemia and indicators related to pancreatic β-cell dysfunction, independently of cortisol. These findings provide new insights into the complex relationship between steroid metabolites and glucose metabolism in patients with NFAI, potentially informing future research on risk assessment and management strategies.

## Data Availability

All datasets generated during the current study are not publicly available but are available from the corresponding author on reasonable request.
